# Osteoporosis in Indian Patients Undergoing Elective Arthroplasty and Spinal Procedures: An Observational Study

**DOI:** 10.7759/cureus.27275

**Published:** 2022-07-26

**Authors:** Dipak Dave, Sujoy K Bhattacharjee, Dilip D Shah, Amrithlal Mascerhans, Paresh C Dey, Subramanian Arumugan, Vikas Mehra, Vinod Agarwal, Sandeep Garg, Satish Chandra Gore, Rajiv Raj Choudhry, Manish Mahajan, Suyash Bharat

**Affiliations:** 1 Orthopedics, Healthcare Global (HCG) Hospital, Ahmedabad, IND; 2 Orthopedics, Sarvodaya Hospital and Research Centre, Faridabad, IND; 3 Orthopedics, Dr. Dilip Shah Clinic, Mumbai, IND; 4 Orthopedics, Vikram Hospital, Bengaluru, IND; 5 Orthopedics, CARE Hospitals, Bhubaneswar, IND; 6 Orthopedics, Bharathirajaa Hospital and Research Centre Pvt Ltd., Chennai, IND; 7 Orthopedics, Dr. Vikas Mehra Orthopedic Centre, Chandigarh, IND; 8 Orthopedics, Lilavati Hospital and Research Centre, Mumbai, IND; 9 Orthopedics, Health City Hospital, Lucknow, IND; 10 Orthopedics, Dr. Satishchandra Gore Spine Clinic, Pune, IND; 11 Orthopedics, Asutosh Hospital, Surat, IND; 12 Pharmacology and Therapeutics, Cadila Healthcare Ltd., Ahmedabad, IND; 13 Pharmacology, Swami Rama Himalayan University (SRHU), Dehradun, IND

**Keywords:** india, fractures, bone density, arthroplasty, osteoarthritis, osteoporosis

## Abstract

Background

This is an observational study conducted to determine the prevalence of osteoporosis and osteopenia in patients undergoing elective arthroplasty and spinal procedures in India.

Methods

This observational, multicentre study included both male and female patients. Their bone mineral density and fracture risk were measured using dual-energy x-ray absorptiometry (DEXA) and Fracture Risk Assessment Tool (FRAX^®^: Centre for Metabolic Bone Diseases, University of Sheffield, UK), respectively, in compliance with the guidelines for Good Epidemiological Practice (ISRCTN: 14543098).

Results

The study revealed that majority (76.4%; 97/127) of the patients had low BMD; over one-third had osteoporosis (39.4%; 50/127) or osteopenia (37%; 47/127). Among those undergoing total knee replacement (TKR)/total hip replacement (THR), majority (75.6%; 59/78) had low BMD (osteoporosis: 38.5% {30/78}; osteopenia: 37.2% {29/78}). Among the patients undergoing spinal procedures, all except two (93.10%; 27/29) had low BMD, two-thirds had osteoporosis (65.5%; 19/29), and around one-fourth had osteopenia (27.6%; 8/29). Radial BMD measurements showed higher prevalence of osteoporosis and osteopenia. Based on FRAX score, nearly 30% of patients were at a high risk of hip fracture in the next 10 years. As per National Osteoporosis Foundation (NOF) guidelines, most (59.79%; 58/97) patients with osteoporosis/osteopenia met criteria for pharmacological treatment.

Conclusions

Regular preoperative bone health evaluation should be adopted and osteoporosis/osteopenia patients should be adequately managed pharmacologically in India.

## Introduction

Osteoporosis, a skeletal disorder characterized by reduced bone strength, increased bone fragility, and a greater predisposition to fractures, is a major global public health problem [1,2]. Over 200 million people worldwide suffer from osteoporosis [3,4]. Factors contributing to vitamin D deficiency, such as genetics, darker skin, vegetarianism, and lactose intolerance, have led to a high prevalence of osteoporosis in India [5]. Approximately 50 million people in India have either osteoporosis or osteopenia, with a greater prevalence among men and postmenopausal women [6-8]. As osteoporosis and osteopenia are known to occur at a relatively young age in the Indian population [9], increasing longevity in Indians may further escalate this mounting threat to wellbeing [1,10].

Osteoporosis and osteoarthritis share common risk factors such as age and female gender; hence they can be present concomitantly, especially in patients with chronic osteoarthritis [11-14]. Osteoporosis is relatively common in patients undergoing total joint arthroplasty (including total knee replacement {TKR}/total hip replacement {THR}) and spinal procedures [15,16]. Traditionally, it was believed that osteoporosis and osteoarthritis have an inverse relationship and patients with advanced osteoarthritis are less likely to develop osteoporosis [12,17-20]. However, newer studies on the association between osteoarthritis and osteoporosis with subsequent fracture risk show conflicting results [21,22]. While some studies show a low prevalence of osteoporosis and increased bone mass in patients with osteoarthritis undergoing TKR/THR [13,22,23], others show a prevalence roughly equal to that in the general population [12,24], while yet others show an increased risk of fractures in these patients [25-28].

Joint replacements and arthroplasty are known to lower bone mineral density (BMD) and increase fracture risk post-surgery [21,28-31]. Moreover, osteoporosis increases the risk of complications, such as aseptic loosening (which leads to nearly half of the indications for the revision of primary arthroplasties), periprosthetic fracture [22, 32], and the complications secondary to implant or bone union failure (including proximal junctional failure and pseudoarthrosis) [16]. In arthroplasty, the bone-implant interface needs to endure high shear stress of physiological loading. Hence, poor quality of peri-implant bone may jeopardize the initial stability of cementless stems and also the long-term success of osseointegration worsening postsurgical outcomes [33,34].

BMD is one of the main determinants of postsurgical outcomes in patients undergoing spine fusion operations with instrumentation [3,35]. Osteopenia and osteoporosis can adversely impact outcomes in elective spine surgery by affecting screw pull-out to fusion rates [36]. As the ability of screws to resist pull-out from bone is directly related to BMD [3,37] an osteoporotic spine considerably lengthens fusion rates, resulting in longer operative time and increased postoperative time for recovery [3,38]. Thus, the consequences of untreated osteoporosis in patients undergoing joint arthroplasty and spinal procedures can be dire, ranging from poor surgical outcomes to increased mortality rates following a fracture [22,36]. As diminishing bone quality and its clinical sequelae continue to escalate in an aging population [36], preoperative evaluation to determine the presence of bone disease, and to assess bone quality is crucial [3,36]. In a survey conducted to map opinions and attitudes of orthopedic surgeons concerning osteoporosis and arthroplasty, around 60% of the orthopedic surgeons stated that low BMD would influence their surgical plan and implant design. However, only 4% of these performed BMD measurements preoperatively [39]. Although dual-energy x-ray absorptiometry (DEXA) is considered to be the "gold standard" in diagnosing osteoporosis, the availability, accessibility, and cost make BMD estimation impractical in a developing country like India [1,40], with approximately 0.26 DEXA machines per million of the general population [41]. The Fracture Risk Assessment Tool (FRAX®: Centre for Metabolic Bone Diseases, University of Sheffield, UK), released by the World Health Organization (WHO) in February 2008, is a web-based algorithm that calculates the 10-year probability of major osteoporosis-related fractures (major osteoporotic joint {MOJ}; clinical vertebral, hip, forearm, or humerus) and hip fractures based on clinical risk factors, and can be useful in fracture prediction in low-resource settings like India [42,43].

Despite several international studies reporting the prevalence of osteoporosis in patients undergoing arthroplasty and spinal procedures [13,22-24,44-48], adequate research on osteoporosis prevalence in Indian patients undergoing these procedures is lacking. While several papers have examined implant design in arthroplasty [13,22-24,47,48], relatively fewer have assessed the quality of the underlying bone into which the implants are inserted [13,22]. Therefore, this study was conducted to determine the preoperative prevalence of osteoporosis and/or osteopenia in Indian patients undergoing prospective elective arthroplasty/spinal procedures and to evaluate their overall bone quality. To the best of our knowledge, this is the first prospective, multicenter study to evaluate these endpoints using both DEXA and FRAX® in Indian patients. Given the recent studies suggesting that homocysteine may be a newly recognized risk factor for osteoporosis, we additionally evaluated the homocysteine levels in these patients and assessed their correlation with osteoporosis/osteopenia [49].

## Materials and methods

This observational, cross-sectional, open-label, multicenter study was conducted as per the principles laid down by the 18th World Medical Assembly, Helsinki, Finland, in 1964 and all applicable amendments thereto and in compliance with the guidelines for Good Epidemiological Practice (ISRCTN 14543098). The study was reviewed and approved by the Sangini Hospital Ethics Committee (ECR/147/INST/GJ/2013/RR-16/1307/2019). Each participating site ensured that all submissions required for approval (e.g., institutional review board/independent ethics committee) were obtained in compliance with local regulations, including the local data protection act. The study design and reporting format followed the recommendations of the Strengthening the Reporting of Observational Studies in Epidemiology (STROBE) guidelines. All participating subjects signed a written informed consent before taking part in the study. Practicing orthopedic surgeons with over 20 years of experience in joint arthroplasty and spinal procedures recruited patients from 11 study centers across India for a period of six months.

Patient selection

Inclusion and Exclusion Criteria

Males ≥60 years and females ≥55 years of age undergoing elective arthroplasty or spinal procedures within a month were included. Subjects who had undergone an elective arthroplasty or spinal procedure in the preceding two years were excluded. Subjects already diagnosed with osteoporosis or treatment therapy for the same were excluded from the study. Also, those on any concomitant chemotherapy drugs were excluded.

Study design and data collection

Data regarding demography, medical and family history, history of orthopedic illness (fractures, secondary osteoporosis, rheumatoid arthritis, and glucocorticoid treatment), smoking status, alcohol consumption, etc. were collected on the case record forms (CRFs).

Before patient enrolment, the investigators reviewed the study protocol. All study data were recorded directly on the CRFs, in which only investigators were authorized to make entries. Patients’ personal data were kept confidential. CRFs or any other documents identified patients by initials and numbers only. All CRFs were checked to ensure they were appropriately filled. Statistical analysis was performed only after all entries were complete and the data were locked.

The data were collected to assess the following primary endpoints - osteopenia (-1> T-score <-2.5) and/or osteoporosis (T-score ≤-2.5) (as defined by National Osteoporosis Foundation {NOF} and American Academy of Family Physicians {AAFP} guidelines) [50,51], >3% 10-year estimated risk of hip fracture (FRAX®), and >20% 10-year estimated risk of major osteoporotic fracture {MOF} (FRAX®).

BMD was measured at the femoral neck and lumbar spine (and radius in a few patients) by DEXA. FRAX® along with BMD/T-score was used to evaluate fracture risk. Blood samples were collected to measure the serum homocysteine levels.

Statistical analysis

The sample size was determined using the following formula: n = p (1-p) x (εα/e)², where "n" represents the required sample size, "p" the estimated proportion of patients with osteoporosis and/or osteopenia among those undergoing elective joint replacement/spinal procedures, εα = 1.96 for α = 5%, and "e" represents the margin of error.

Assuming that 20% of the patients undergoing elective replacement/spinal procedures have a T-score of ≤-1 (based on DEXA), >3% 10-year estimated risk of hip fracture, >20% 10-year estimated risk of major osteoporotic fracture (MOF) and homocysteinemia, to determine the prevalence of osteoporosis and/or osteopenia with an absolute precision of 10% at a 95% confidence interval (CI), a sample size of 62 patients was required. Assuming a drop out of 10%, the requirement was around 69.

All data recorded were summarized using descriptive analyses. Mean standard deviation (SD), median, and range (min-max) were used to describe continuous variables. Frequency and percentage (two-sided 95% CI) were used to present categorical variables. Statistical analyses were performed using the SPSS software (Armonk, NY: IBM Corp.).

## Results

Demographics and clinical characteristics

The mean age of included patients was 66.83±7.9 years. The majority (76.4%; 97/127) were women. Two-thirds (66.9%; 87/127) had above-normal BMI and were classified as overweight, obese, or morbidly obese. Most patients were scheduled to undergo TKR/THR (61.4%; 78/127) and fewer were scheduled to undergo spinal procedures (22.8%; 29/127) (Table 1).

**Table 1:** Characteristic of patients undergoing elective spinal and arthroplasty procedures at baseline assessment. All values indicate n (%) other than those specified. *Patients where the category of procedure (TKR/THR/spinal) was not specified. TKR: total knee replacement; THR: total hip replacement

Baseline characteristics			Values
Age (in years)	Mean±SD		66.83±7.9
Gender		Female	97 (76.4)
		Male	30 (23.6)
Weight (kg)		Female	68.20
		Male	66.00
		Total subjects, mean±SD	67.10±12.44
Height (cm)		Female	152.11
		Male	157.45
		Total subjects, mean±SD	154.78±8.14
BMI (in classes)		Underweight (<18.5)	2 (1.6)
		Normal (18.5-24.9)	40 (31.5)
		Overweight (25-29.9)	42 (33.1)
		Obese II (30-34.9)	29 (22.8)
		Obese II (35-39.9)	12 (9.4)
		Morbid obese (>40)	2 (1.6)
		Mean±SD	28.06±5.17
Surgical procedure planned		Total knee replacement	74 (57.8)
		Unilateral	47 (36.7)
		Bilateral	27 (21.1)
		Total hip replacement	4 (3.1)
		Spinal procedures	29 (22.8)
		Unspecified*	20 (15.7)

Proportion of patients having osteopenia/osteoporosis

The majority of patients (76.4%; 97/127) had low BMD, over one-third had osteoporosis (39.4%; 50/127) or osteopenia (37%; 47/127), and less than one-fourth (23.6%; 30/127) had normal BMD (Table 2). Among those undergoing TKR/THR, the majority (75.6%; 59/78) had low BMD (osteoporosis: 38.5% {30/78}, osteopenia: 37.2% {29/78}). Of the patients undergoing spinal procedures, all but two (93.10%; 27/29) had low BMD, two-thirds had osteoporosis (65.5%; 19/29), and around one-fourth had osteopenia (27.6%; 8/29) (Table 2). Most (79.01%; 64/81) patients showed evidence of low BMD at the radial bone, of which nearly half (45.68%; 37/81) had osteoporosis, and one-third (33.33%; 27/81) had osteopenia, and only one-fifth (20.99%; 17/81) had normal BMD (Table 2).

**Table 2:** Prevalence of osteopenia/osteoporosis in patients undergoing procedures. *Patients where the category of procedure (TKR/THR/spinal) was not specified. TKR: total knee replacement; THR: total hip replacement

Proportion of patients (n=127)	Normal (n=30)	Osteopenia (n=47)	Osteoporosis (n=50)	p-Value (chi-square)
Arthroplasty/spinal surgery	30 (23.6)	47 (37)	50 (39.4)	0.001*
TKR/THR	19 (24.4)	29 (37.2)	30 (38.5)	
Spinal procedure	2 (6.9)	8 (27.6)	19 (65.5)	

Overall bone quality

BMD and T-score

Mean BMD at the femoral neck was lower than that observed at the spine (osteoporosis group: 0.68±0.13 vs 0.85±0.15; osteopenia group: 0.82±0.08 vs 1.05±0.18) (Table 3). The mean T-score was lower at the radius than at the femoral neck and spine in patients with osteoporosis (radius: -3.50±1.34; femoral neck: -2.64±0.89; spine: -2.85±1.2), as well as in those with osteopenia (radius: -1.79±1.76; femoral neck: -1.56±0.5; spine: -1±1.01). In the osteoporosis group, the mean T-score (-3.50±1.34) at the radius was indicative of severe osteoporosis (Table 3).

**Table 3:** Assessment of overall bone quality in patients undergoing elective spinal and arthroplasty procedures. *P-value is significant. **Patients where the category of procedure (TKR/THR/spinal) was not specified. BMD: bone mineral density; FRAX: Fracture Risk Assessment Tool; MOF: major osteoporotic fracture

Bone quality parameter		Normal BMD (n=17)	Osteopenia (n=27)	Osteoporosis (n=37)	p-Value (ANOVA)
BMD (mean±SD)	Femoral neck	1.03±0.12	0.82±0.08	0.68±0.13	0.000*
	Vertebra	1.21±0.16	1.05±0.18	0.85±0.15	0.000*
T-score (mean±SD)	Femoral neck	0.23±0.9	-1.56±0.5	-2.64±0.89	0.000*
	Vertebra	0.61±1.25	-1±1.01	-2.85±1.2	0.000*
	Radius	-0.76±1.51	-1.79±1.76	-3.50±1.34*	0.000**
FRAX score (mean±SD)	MOF	4.58 (±3.1)	6.74 (±5.39)	9.21±5.25	0.001*
	Hip fracture	0.90 (±1.16)	2.17 (±3.24)	4.15±3.43	0.001*

FRAX Score

**Figure 1:**
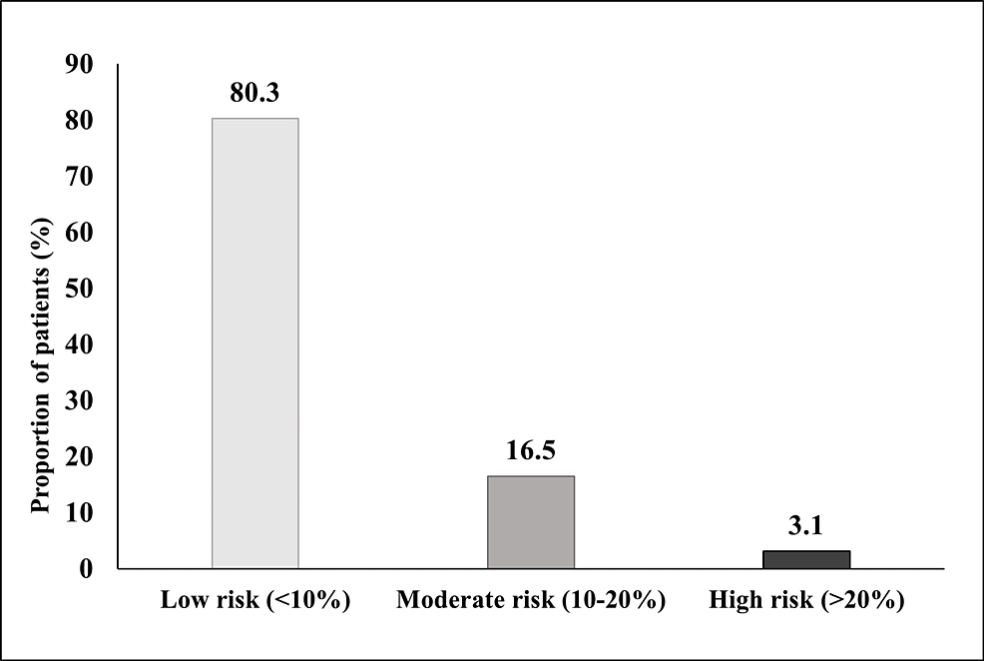
Assessment of 10-year fracture risk of major osteoporotic fracture.

**Figure 2:**
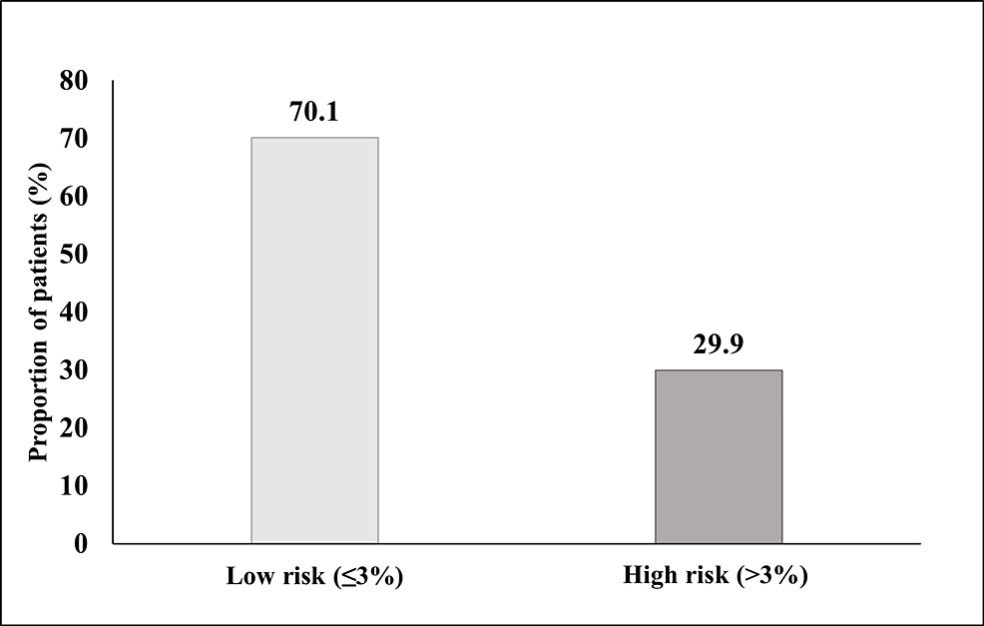
Assessment of 10-year fracture risk of hip fracture.

In patients with osteoporosis, the mean 10-year probability of hip fracture and MOF was 4.15±3.43 and 9.21±5.25, respectively (Table 3). Nearly 30% (29.9%; (38/127) of the patients showed a high (>3%) 10-year estimated risk of hip fracture; one-fifth (19.68%; 25/127) had a moderate (10-20%) to high (>20%) 10-year estimated risk of MOF (Figures 1, 2).

Serum Homocysteine Levels

Serum homocysteine levels were 13.79±4.14 mcmol/L, 17.12±11.7 mcmol/L, and 15.19±8.52 mcmol/L in the osteoporosis, osteopenia, and normal BMD groups, respectively. Serum homocysteine values did not show any correlation with BMD, T-scores, or BMI.

Pharmacological Treatment

Pharmacotherapy should be guided by the presence/absence of vertebral/hip fractures, or the severity of risk based on clinical factors, although bisphosphonates remain the first choice in most cases. Anabolic agents and newer antiresorptive like denosumab are to be added to high-risk individuals as per ISBMR guidelines [52]. Regular follow-up is essential to ensure adherence and response to therapy. Almost 60% (59.79%; 58/97) of the study participants with osteoporosis and osteopenia met the criteria for pharmacological treatment, as per the NOF guidelines.

## Discussion

This first multicenter, prospective, cross-sectional study of patients undergoing arthroplasty and spinal procedures in India demonstrated a high prevalence of osteoporosis. Due to the lack of research and literature in this area in Indian population, this study was an effort to fill the lacunae. In this study, the majority of the patients undergoing TKR/THR had low BMD, osteoporosis prevalence rates were higher in those undergoing spinal procedures. BMD measurements at the radius revealed higher osteoporosis rates and lower mean T-scores than those estimated from femoral neck and spine BMD values. Nearly 30% of the patients had a high (>3%) 10-year estimated risk of hip fracture. No correlation was observed between serum homocysteine values and BMD, T-scores, or BMI. Almost 60% of the patients with osteoporosis and/or osteopenia met the criteria for pharmacological treatment, as per the NOF guidelines.

Our study observed that as high as 76.4% of the patients undergoing arthroplasty or spinal procedures had low BMD, over one-third had (39.4%) osteoporosis, and an almost equal proportion (37%) had osteopenia. In patients undergoing TKR/THR, a similar prevalence (75.6%) of low BMD presenting as either osteoporosis (38.5%) or osteopenia (37.2%) was noted. These findings were substantiated by a retrospective case series of 200 patients undergoing TKR/THR at a single tertiary-care center that showed evidence of low bone mass in 86% of the patients, with osteoporosis prevalent in one-third (33%) of them [15]. In another prospective study between October 2009 and November 2014, which evaluated 107 Japanese postmenopausal women (aged ≥55 years) undergoing TKR, 70% of patients had low BMD, with an osteoporosis prevalence ranging between 10% and 18%, based on the site of BMD evaluation [13].

In terms of the site of the BMD evaluation, our study noted some variation in BMD and T-score values at different sites e.g., the mean BMD at the femoral neck was lower than that noted at the spine. T-score measurements at the radial bone revealed a higher incidence (79.01%; 64/81) of low bone mass and a higher incidence of osteoporosis (45.68%; 37/81). In other words, the noted prevalence of osteoporosis and osteopenia increased when radial bone BMD was examined. Similar results were noted in a cross-sectional observational study conducted at the tertiary referral center in Newcastle upon Tyne, UK. Of the 199 patients (aged between 65 and 80) with osteoarthritis awaiting TKR/THR, osteoporosis was more commonly detected in the forearm (14%) and a stronger correlation was noted between the BMD measurements of the forearm and proximal femur (r=0.78 for both). Based on these findings, Lingard et al. strongly emphasized the importance of measuring forearm BMD for the detection of osteoporosis in osteoarthritis [48]. The presence of osteophytes, especially in the lumbar spine, elevates BMD measurements at these sites, resulting in inaccurately high BMD values when the anteroposterior spine projection is used [44,48]. Likewise, the presence of bone sclerosis can lead to a higher BMD in an osteoarthritic hip than in the contralateral hip. Hence, it is recommended that in patients with osteoarthritis, bone densitometry should always include other non-affected, distant anatomical sites (such as the forearm), to prevent the overestimation of BMD in these patients [47].

The diagnosis of concomitant osteoporosis in a significant proportion of patients with end-stage osteoarthritis may be missed unless BMD measurements are performed at sites distant from the joints affected by osteoarthritis [48]. The mean radial T-score observed in our study was lower than that noted in the femoral neck and the spine in both osteoporosis and osteopenia groups. In the osteoporosis group, the mean radial T-score (-3.50±1.34) was indicative of severe osteoporosis. Patients with osteoporosis showed high mean FRAX scores for hip fracture; nearly 30% of them were at a high (>3%) 10-year estimated risk of hip fracture. Deterioration in bone microstructure plays an important role in the pathogenesis of fractures. Considering the protective effect of weight-bearing on bone microstructure, the distal radius bone microarchitecture seems to be a better indicator of vertebral fragility than the bone architecture at the distal tibia. In patients with severe vertebral deformity, deficits in the cortical and trabecular compartments of the distal radius are particularly pronounced [3]. On the other hand, the femoral neck, most commonly used for the measurement of BMD, can detect low bone mass in relatively fewer patients with spinal pathology [44].

In our study, patients undergoing spinal procedures had a relatively higher prevalence (93.10%) of low bone mass, predominantly (65.5%) presenting as osteoporosis. The prevalence was in line with that noted in two other retrospective studies, conducted at two different time points. In the retrospective, observational study conducted at Yongdong Severance Hospital, South Korea, between January 2005 and December 2005, of the 446 patients older than 50 years who underwent spine procedures, 77.57% (346/446) showed evidence of low BMD, including 207 (46.4%) patients with osteopenia and 139 (31.1%) with osteoporosis [46]. In the other retrospective review conducted at Mayo Clinic, Rochester, USA, between 2007 and 2014, of 140 patients undergoing spinal surgery, 68.57% (96/140) of the patients showed evidence of low bone mass, comprising 58.57% (82/140) with osteopenia, and 10% (14/140) with osteoporosis [45].

In our study, no correlation was observed between the serum homocysteine values and BMD, T-scores, or BMI. Two other studies reinforced this finding, thus demonstrating that serum homocysteine levels were not associated with BMD at either the femoral neck or the lumbar spine and that the associations between the homocysteine levels and fracture risk noted in previous studies were independent of BMD [53,54].

The NOF guidelines recommend pharmacological treatment of osteoporosis in patients with a T-score <-2.5 at the femoral neck or spine, previous history of hip or vertebral fracture, a T-score between -1 and -2.5 at the femoral neck or spine, and 10-year risk of hip fracture >3% or MOJ >20% [51]. As per these guidelines, around 60% of the patients with osteoporosis and osteopenia in our study met the criteria for receiving pharmacological treatment. The guidelines of the American Association of Clinical Endocrinologists (AACE) strongly recommend pharmacological therapy not only for patients with osteoporosis (T-score of -2.5 or lower in the spine, femoral neck, total hip, or 1/3 radius) but also for those with osteopenia (T-score between -1.0 and -2.5), if the 10-year probability for MOJ is ≥20% or the 10-year probability of hip fracture is ≥3% (as measured by FRAX®), or if osteopenia patients have a history of a fragility fracture of the hip or spine. The guidelines emphasize the assessment of clinical fracture risk using FRAX® for the initial evaluation of osteoporosis. In most patients, treatment is initiated because of the high fracture risk. As the level of fracture risk may influence the selection of the initial treatment, the AACE guidelines recommend the stratification of patients by the level of fracture risk at the time of treatment initiation. Moreover, a very high fracture risk may require more aggressive treatment to reduce it to an acceptable level. In patients with very high fracture risk, anabolics may be preferable as initial therapy. As anabolics stimulate bone formation (through increased osteoblastic activity) and restore degraded bone microarchitecture, they are expected to have greater effects on BMD and fracture reduction than antiresorptive therapies (which merely inhibit bone breakdown) [21,52]. The superiority of anabolics over antiresorptive agents in reducing vertebral fracture risk in very high fracture risk patients has been observed in several studies [52].

This study provides valuable country-specific data and crucial insights about this patient population. Since the quality of the underlying bone is an important determinant of postsurgical outcomes in patients undergoing joint arthroplasty and spinal procedures, the study findings may have substantial implications for preoperative planning and evaluation. The study met the required sample size (127 patients included vis-à-vis 69 required) which further validates the results. Moreover, as patients were recruited from 11 centers across India, the study population was adequately representative of the geographical diversity across the country, thus enhancing the validity and generalizability of the results.

However, being an observational study, it is subject to certain inherent limitations such as bias, confounding factors, etc. The cross-sectional design did not permit the long-term follow-up of patients to observe the impact of low bone mass on postoperative outcomes. Though the study did capture sub-optimal bone health in most patients, it did not objectively scrutinize the management strategies employed by orthopedic surgeons to tackle this problem.

## Conclusions

This cross-sectional study indicates that a sizable proportion of patients undergoing joint arthroplasty or spinal procedures in India have osteoporosis or osteopenia. It was also noted that the prevalence of osteoporosis and osteopenia increased when radial bone BMD was examined. It can also be concluded that no correlation was observed between the serum homocysteine values and BMD, T-scores, or BMI in patients of this study. However, it is crucial that these risk factors are detected early for the patients to qualify for pharmacological treatment as per the guidelines. Hence, considering the detrimental impact of osteoporosis on recovery and postsurgical outcomes, it becomes imperative to encourage preoperative screenings and conduct a thorough bone health evaluation in all patients undergoing joint arthroplasty and spinal procedures.
